# An Effective Fluorescent Marker for Tracking the Dispersal of Small Insects with Field Evidence of Mark–Release–Recapture of *Trissolcus japonicus*

**DOI:** 10.3390/insects15070487

**Published:** 2024-06-29

**Authors:** Ryan L. Paul, James R. Hagler, Eric G. Janasov, Nicholas S. McDonald, Saliha Voyvot, Jana C. Lee

**Affiliations:** 1 Department of Horticulture, Oregon State University, Corvallis, OR 97331, USA; mcdonnic@oregonstate.edu (N.S.M.);; 2 Crops Disease and Pest Management Research Unit, USDA-ARS, Corvallis, OR 97330, USAjana.lee@usda.gov (J.C.L.); 3 Arid-Land Agricultural Research Center, USDA-ARS, Maricopa, AZ 85138, USA; james.hagler@usda.gov; 4 Ege Forestry Research Institute, General Directorate of Forestry, Izmir 35040, Turkey

**Keywords:** marking, mark–release–recapture, dispersal, parasitoid, *Pachycrepoideus vindemiae*, *Ganaspis brasiliensis*

## Abstract

**Simple Summary:**

Understanding insect dispersal helps us predict the spread of insect pests and their natural enemies. Dispersal can be studied by marking, releasing, and recapturing insects, known as mark–release–recapture (MRR). MRR techniques should be convenient, economical, and persistent. Currently, there are few options for marking small insects, such as parasitic wasps, that do not negatively affect their natural behaviors. We evaluated a marker used as a trap for high-value property (such as bank vaults) for its effectiveness in MRR research with parasitic wasps. This marker is easy to apply and can be detected visually using UV light. We tested this marker on a pest fruit fly and three parasitic wasp species that are used in biological control. We found that the marker lasted for the lifespan of all the insects tested and had little impact on the survival, parasitism, flight, or activity patterns of the wasp species. We were also able to recapture marked wasps up to three weeks after release and 100 m away from the release site, indicating that this technique can be used to track the dispersal of small wasps.

**Abstract:**

Understanding insect dispersal helps us predict the spread of insect pests and their natural enemies. Dispersal can be studied by marking, releasing, and recapturing insects, known as mark–release–recapture (MRR). MRR techniques should be convenient, economical, and persistent. Currently, there are limited options for marking small parasitoids that do not impact their fitness and dispersal ability. We evaluated commercially available fluorescent markers used in forensics. These fluorophores can easily be detected by ultraviolet (UV) light, requiring minimal costs and labor to process the marked specimens. This fluorophore marking technique was evaluated with the pest *Drosophila suzukii* and three parasitoids: *Trissolcus japonicus*, *Pachycrepoideus vindemiae*, *Ganaspis brasiliensis* (=*G. kimorum*). We evaluated the persistence of the marks on all the insects over time and examined the parasitoids for impacts on longevity, parasitism, locomotor activity, and flight take-off. The green fluorophore marker persisted for over 20 days on all four species. Marking generally did not consistently reduce the survival, parasitism rate, locomotor activity, or take-off of the parasitoids tested. Marked *T. japonicus* were recaptured in the field up to 100 m away from the release point and three weeks after release, indicating that this technique is a viable method for studying parasitoid dispersal.

## 1. Introduction

Dispersal is important to understanding population dynamics and the spread of invasive insects and natural enemies [1,2,3,4]. Dispersal traits can directly impact the establishment of introduced species [1], management strategies for pest insects [5,6], or the effectiveness of biological control agents [3,7,8]. Dispersal studies are key to determining the proper number and location of release sites for biological control agents [3,7,9]. While laboratory studies can determine characteristics such as flight capacity [10], examining insects’ dispersal ability in the field is critical. Yet, this can be challenging for tiny insects, including many parasitoids implemented for biological control.

Several techniques have been used for marking insects to track their dispersal in the field including dyes, paint tags, mutilation, trace elements, and proteins [11,12,13,14,15,16,17]. These markers vary in their ease of application, detection, and efficacy [13]. Methods for marking tiny parasitoids are more limited. Dyes and powders are inexpensive but often not persistent, and their weight can impact survival and flight [13,18,19]. Rare or trace elements have proven effective but require complex, time-consuming analysis to detect markers [13]. Many of these issues have been addressed using protein-specific immunoassays that are sensitive to trace amounts of protein, easy to apply, and inexpensive for large-scale use without negative impacts on delicate parasitoids [16].

Although protein immunomarking has been used effectively in the field with parasitoids [16,18,20], each individual must be analyzed for the marker protein by an enzyme-linked immunosorbent assay (ELISA). The ELISA procedure is simple, but it does require some training and specialized equipment. Also, specimens must be removed from sticky traps, which are commonly used for capturing parasitoids. This process adds labor and increases the potential for some individuals to be missed when inspecting traps.

Recently, a new liquid fluorophore taggant has shown effectiveness for marking a variety of insects. It is inexpensive and easy to apply and can be visually detected [12,21]. SmartWater^®^ (SmartWater CSI, Fort Lauderdale, FL, USA) is a fluorescent liquid marketed primarily as a trap for marking criminals attempting to access valuable or secure property (e.g., bank vaults). As such, it is non-toxic, invisible in natural lighting but viewable with UV light, persistent under field conditions, and available in multiple colors. It has been demonstrated to effectively mark a wide range of insect taxa, including small, delicate insects such as mosquitoes and whiteflies [12,21,22], but to date, it has not been tested for use on parasitoids.

We conducted a series of experiments to determine the long-term persistence and fitness impacts of SmartWater^®^ fluorophores (hereafter referred to as cartax green or magenta) for marking multiple species of small parasitoids. We assessed the viability of the marker visually over the lifespan of three parasitoid species, *Trissolcus japonicus* (Ashmead) (Hymenoptera: Scelionidae), *Pachycrepoideus vindemiae* (Rondani) (Hymenoptera: Pteromalidae), and *Ganaspis brasiliensis* (Ihering) (=*G. kimorum* Buffington [23]; Hymenoptera: Figitidae) plus spotted-wing drosophila, *Drosophila suzukii* Matsumura (Diptera: Drosophilidae). For each parasitoid species, we also compared the longevity, flight ability, locomotor activity, and parasitism ability between marked and unmarked individuals. Finally, we demonstrate that fluorophores can be an effective, long-lasting, and easy-to-assess marker for field mark–release–recapture (MRR) studies of small parasitoids.

## 2. Materials and Methods

### 2.1. Insects

All insects were obtained from laboratory-reared colonies. *Trissolcus japonicus* were reared on 1 to 3-day-old *Halyomorpha halys* egg masses placed in 50 mL plastic vials containing streaks of honey on the side and topped with cotton plugs. *Halyomorpha halys* were reared in 31 × 31 × 31 cm mesh cages (BugDorm-1, MegaView, Taipei, Taiwan) with green beans, carrots, and grapes changed twice a week and fava bean plants and peanuts/sunflower seeds changed biweekly. Egg masses were collected daily on weekdays and either used for parasitism or frozen for further use. *Pachycrepoideus vindemiae* were reared on *Drosophila suzukii* pupae as described in Hogg et al. [24]. *Ganaspis brasiliensis* were reared on *D. suzukii* larvae-infested blueberries rotated every 2–3 d through 11 × 7 cm plastic containers containing 15–20 wasps, or 25 × 25 × 27 cm plastic cages containing 30+ wasps [25,26]. All newly emerged parasitoids were kept in plastic 50 mL vials and fed pure honey prior to use in the experiments. *D. suzukii* were reared in cages and on an agar–yeast diet as described in Woltz et al. [27].

### 2.2. Marking Procedure

Up to 200 adult insects were placed into a 946 mL plastic deli cup for marking. Insects were chilled at 4 °C for up to 30 min before transferring them into the cup. All marking was performed in a fume hood using a medical nebulizer (Micro Mist Handheld Nebulizer, Teleflex LLC, Morrisville, NC, USA) inserted into a 2.5 cm hole in the side of the cup, with fine mesh covering the nebulizer opening. Parafilm was used to seal the opening at the opposite end of the nebulizer T-adapter. A mesh opening at the top of the cup allowed excess nebulized marker to escape. The nebulizer was attached to a laboratory air supply, and 2 mL of fluorophore (SmartWater^®^; SmartWater CSI, Fort Lauderdale, FL, USA) was nebulized into the cup under a fume hood. After marking, parasitoids were air-dried for 30 min. All insect species were marked with cartax green fluorophore, and then a second set of *T. japonicus* and *P. vindemiae* were marked with magenta fluorophore for comparison. Due to the differences in optimal mixing ratios of fluorophore and polymer, fluorophore solutions for each color were prepared according to Hagler et al. [21]. For the 2023 field-released insects, wasps were double-marked using the same methods with 2 mL of undiluted chicken or rabbit IgG blood serum in addition to the 2 mL of cartax green. The serum protein-marked insects allowed the tracking of dispersal from multiple locations since there were two protein marks available, while the fluorophore mark provided an easy-to-identify mark to further assay by the rabbit- and chicken-specific ELISAs described by Hagler [28].

### 2.3. Experiment 1: Marking Persistence

Marking persistence was tested for all four insects in the study: *D. suzukii*, *T. japonicus*, *P. vindemiae*, and *G. brasiliensis*. Marked insects were maintained in mesh cages (24 × 24 × 24 cm BugDorm 42222, MegaView, Taichung, Taiwan). Each cage contained a 29 mL cup with a water wick and a separate food source. *Trissolcus japonicus* were given a smear of pure honey, which was replaced as needed. *Pachycrepoideus vindemiae* and *G. brasiliensis* were provided 50% honey–water soaked in cotton in a 55 mm Petri dish, which was moistened every 2–3 d and replaced weekly. *Drosophila suzukii* were given an artificial diet [27], replaced weekly. Folded cardstock was placed in cages for insects to hide under. All insects were marked in batches depending on colony availability. A subset of each group of marked insects was removed and frozen at −20 °C every other day to check for marker presence starting at day 0 after air-drying from marking and ending at day 22 for parasitoids or day 30 for fruit flies. Each individual was observed under a stereo microscope using a fluorescence viewing system with a 360–380 nm UV LED (SFA-UV, Nightsea, Lexington, MA, USA). Parasitoids were scored on a scale from 0 to 5 based on the amount of area covered by the marker on six body regions (head, thorax, abdomen, wings, antennae, and legs). The proportion of body covered was visually estimated using groupings of 0, 1–5, 6–19, 20–49, 50–79, and 80–100% and then assigned a corresponding rank of 0, 1, 2, 3, 4, or 5, respectively. These categories were based on categories that were easily separable by visual observers.

### 2.4. Experiment 2: Longevity Experiment

A group of recently emerged (0–2 d old) marked and unmarked parasitoids were each placed into separate mesh cages (24 × 24 × 24 cm BugDorm 42222, MegaView, Taiwan). Each cage contained a 29 mL cup with a water wick and honey or artificial diet. Cages were checked daily, and dead insects were recorded and removed until none remained. Several batches of each insect species were set up on different days to reach full replication (*T. japonicus*: *n* = 88 marked, 60 unmarked; *P. vindemiae*: *n* = 42 marked, 57 unmarked; *G. brasiliensis*: *n* = 53 marked, 90 unmarked).

### 2.5. Experiment 3: Parasitism

A single marked or unmarked female wasp was placed in a small container with hosts and provided honey. Specifically, *T. japonicus* was placed in a 10-dram plastic snap-cap vial with a single *H. halys* egg mass, *P. vindemiae* in a 10-dram vial with a *D. suzukii* pupa, and *G. brasiliensis* in a 29 mL plastic cup with two blueberries infested with *D. suzukii* 1st instar larvae. *Trissolcus japonicus* and *P. vindemiae* were removed after 7 d, and *G. brasiliensis* after 3 d. The presence of fluorescent marker was confirmed among marked individuals. Hosts were reared for 3–4 weeks until emergence, and the proportion of emerging parasitoids was recorded. Unemerged hosts were dissected to look for undeveloped parasitoids. Wasps were marked on 3–4 separate days to achieve full replications (*T. japonicus n* = 21 marked, 26 unmarked; *P. vindemiae n* = 26 marked, 44 unmarked; *G. brasiliensis n* = 30 marked, 30 unmarked).

### 2.6. Experiment 4: Flight Ability

*Trissolcus japonicus* and *P. vindemiae* were tested for flight take-off from a point surrounded by a moat. A 90 mm Petri dish was placed into the center of a 142 mm Petri dish and weighed down with a closed 37 mL cup filled with sand. The larger Petri dish was then filled with water until the water level just met the top of the smaller dish. Approximately 20 parasitoids were placed at a time into the center of the small dish and observed until all individuals had attempted to leave or after 24 h using a camera. Since parasitoids were unable to cross the water by walking, the number successfully leaving the smaller Petri dish (without drowning) was recorded to determine flight success and compared between marked and unmarked parasitoids. Three separate days of take-offs were set up for each parasitoid (*T. japonicus*: *n* = 44 marked, 39 unmarked; *P. vindemiae*: *n* = 60 per treatment).

### 2.7. Experiment 5: Activity Monitor

Marked and unmarked *T. japonicus*, *P. vindemiae*, and *G. brasiliensis* parasitoids were placed individually into 25 mm diam. glass vials with cotton plugs on each end and placed into a locomotor activity monitor (LAM25H, Trikinetics, Waltham, MA, USA). These monitors record how many times each individual crosses an infrared beam. Activity monitors were set up as described in Paul et al. [29] but kept in an environmental growth chamber at 25 °C, 50% RH, and a 16:8 L:D photoperiod. Wasps were randomly assigned one of the 64 positions between two activity monitors (32 slots each). Each vial contained a small dab of honey at both ends. Parasitoids were left in the monitor for 9 d. The first and last partial days of recording were removed from analysis to yield 7 d of full activity data. Individuals who died before 7 d were omitted from the analysis.

### 2.8. Field Mark–Release–Recapture

During the summer of 2022 and 2023, *T. japonicus* were marked as described above. In 2021, approximately 9000 marked wasps were released weekly at a hazelnut farm near Millersburg, OR at a single site from July through September. The number of individuals released each week varied from several hundred to ~2000. Yellow sticky traps (*n* = 53) were placed around the farm every 50 m in each cardinal direction and diagonal from the release site (except N, where the farm edge was) and every five trees (for the first 20 trees) from the east and west edges of the farm (see Figure S1). Traps were collected weekly through four weeks following the final release.

In 2023, wasps were released at a mixed hazelnut and apple farm. *Trissolcus japonicus* were first marked with blood serum protein (rabbit or chicken IgG) and then marked with cartax green fluorophore using the method described above. Wasps were released at two locations, with those marked by rabbit IgG released at the center of the field between the two crops (Figure S2) and chicken IgG-marked wasps released at the intersection of the field edge and riparian zone (Figure S3; ~9500 wasps released per treatment). Yellow sticky traps were placed in each cardinal direction and diagonally every 25 m for the first 100 m and then every 50 m after to the edge of the field or property (150 m maximum) for a total of 51 traps. Releases and trap collection took place once a week from July through October (last release September 21).

### 2.9. Statistical Analysis

All analyses were performed with R version 4.2.2. The longevity of marked versus unmarked parasitoids was analyzed using the “survminer” package in R to perform a log-rank test between the survivorship of marked and unmarked treatments. Proportions of parasitized hosts exposed to *T. japonicus* and *G. brasiliensis* were analyzed as binomial counts using logistic regression with logit link (binomial family for glm function in R) and marking treatment as the predictor. Parasitism of hosts exposed to *P. vindimiae* was analyzed using a chi-square test since wasps were exposed to hosts individually. Any parasitism replicates where no wasps or hosts emerged were removed from the analysis. Analysis of flight success was also performed using a chi-square test. A custom script was developed using the “Rethomics” package in R [30] for analyzing activity monitor data. Linear models were used to compare total daily, duration, and activity intensity between marked and unmarked treatments for each parasitoid on each experimental day.

## 3. Results

### 3.1. Experiment 1: Marking Persistence

**Figure 1:**
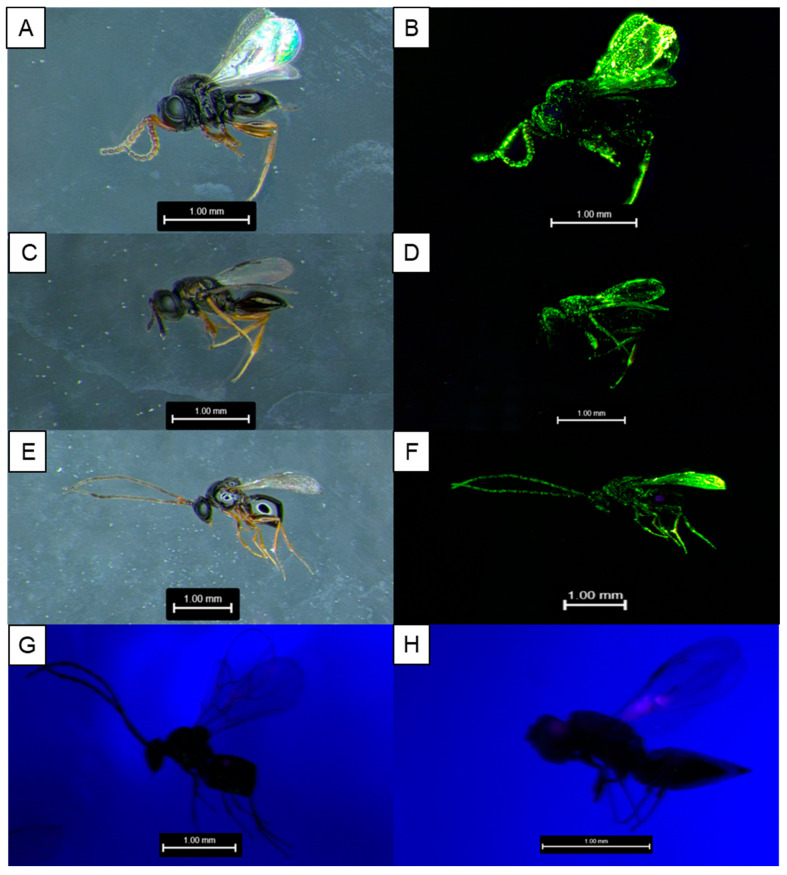
Cartax green fluorophore-marked specimens of *T. japonicus* (**A**,**B**), *P. vindemiae* (**C**,**D**), and *G. brasiliensis* (**E**,**F**) under normal (**A**,**C**,**E**) and UV light (**B**,**D**,**F**). Unmarked parasitoids do not show green fluorescence under UV light (**G**,**H**).

Cartax green marker was visually observable over the entire lifespan of every insect species tested (Figure 1). Maximum days marked differed only based on the longevity of individual species (*T. japonicus* = 22 d; *P. vindemiae*, *G. brasiliensis* = 22 d; *D. suzukii* = 30 d). The proportion of the body covered decreased significantly with days since marking (χ^2^ = 238.0, df = 1, *p* < 0.001; Figure S4), and three individual *G. brasiliensis* after 20 days were the only specimens without observable marks. Marks were typically only visible on a small area of the body after the first several days, and the location differed between species (Figures S5–S8). The abdomens retained marks poorly in all species, while the thorax remained consistently marked in nearly all species except *G. brasiliensis*. Individual *G. brasiliensis* retained the marker most consistently on the head.

### 3.2. Experiment 2: Survival

**Figure 2:**
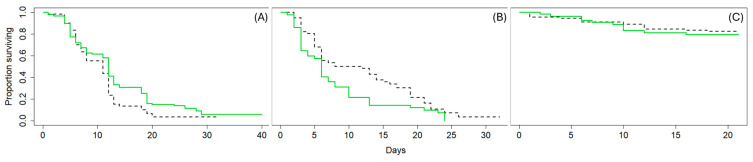
Proportion of surviving marked and unmarked (**A**) *Trissolcus japonicus*, (**B**) *Pachycrepoideus vindemiae*, and (**C**) *Ganaspis brasiliensis*. Plots are survival curves depicting the proportion of the original population remaining (*y*-axis) on each day of the experiment (*x*-axis). Marked wasps are depicted in solid green and unmarked wasps in dashed black lines.

Overall, the effects of marking on parasitoid fitness were minimal. Marked *T. japonicus* survived longer than unmarked wasps (χ^2^ = 4.5, df = 1, *p* = 0.03, Figure 2A), whereas *P. vindemiae* survived longer when unmarked (χ^2^ = 5, df = 1, *p* = 0.03, Figure 2B) and *G. brasiliensis* survival did not differ with marking (χ^2^ = 0.2, df = 1, *p* = 0.7, Figure 2C).

### 3.3. Experiment 3: Flight Ability

Marking with cartax green fluorophore had no effect on the flight ability of parasitoids. All wasps successfully left the arena except for three *T. japonicus*, which drowned in the water. Although *P. vindemiae* were slower to leave the arena overall, all individuals still successfully left the arena in both treatments.

### 3.4. Experiment 4: Parasitism

**Figure 3:**
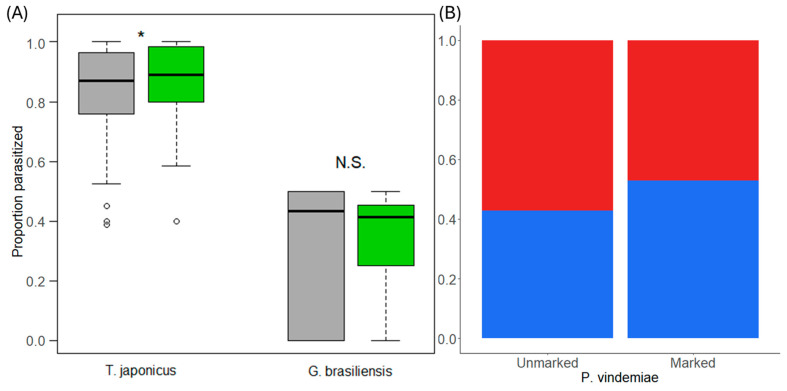
(**A**) Proportion of hosts parasitized by unmarked and marked female *T. japonicus* and *G. brasiliensis*. Boxes represent the 1st and 3rd quartiles, and the line represents the median of the parasitism proportion with whisker ends displaying the range of data plus outliers as individual dots. Gray boxes represent unmarked wasps and green boxes are marked wasps. Proportions are based on the ratio of parasitized to unparasitized hosts exposed to each individual parasitoid. Significant differences are marked above by * and N.S. denotes non-significant differences. (**B**) Proportion of hosts parasitized by unmarked and marked *P. vindemiae* females. Each column shows the proportion of individual hosts that were parasitized (blue) or unparasitized (red). Each female was exposed to only a single host pupa.

Marked *T. japonicus* parasitized more hosts than unmarked *T. japonicus* (χ^2^ = 3.96, df = 1, *p* = 0.046, Figure 3A). Unmarked *T. japonicus* parasitized on average 81.5 ± 3.7% (mean ± standard error) of available hosts per egg mass, while marked females parasitized 84.9 ± 3.7%. Both other parasitoid species were able to parasitize equally regardless of marking treatment. Unmarked *P. vindemiae* parasitized 18/42 (42.9%) of hosts while marked females parasitized 18/34 (52.9%) (χ^2^ = 0.77, df = 1, *p* = 0.381, Figure 3B). Unmarked *G. brasiliensis* parasitized 33.08 ± 3.4% of available hosts per replicate while marked females parasitized 34.14 ± 4.3% on average (χ^2^ = 0.65, df = 1, *p* = 0.419, Figure 3A).

### 3.5. Experiment 5: Locomotor Activity

**Figure 4:**
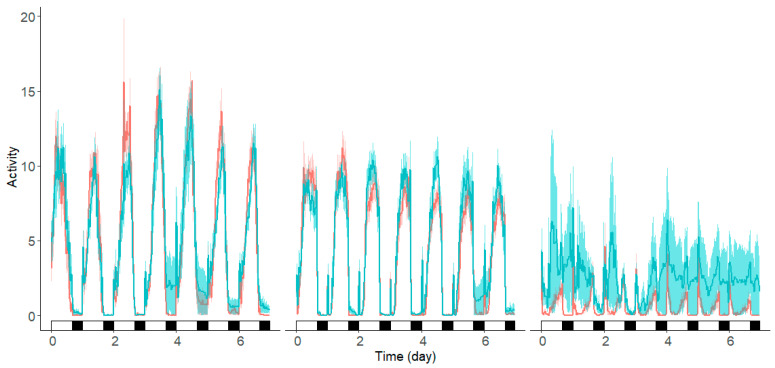
Locomotor activity patterns of marked and unmarked *T. japonicus* (**left**), *P. vindemiae* (**middle**), and *G. brasiliensis* (**right**). Curves show average activity readings across with shading for standard error. Marked wasps are shown in green and control wasps in red. At the bottom of each plot, lights on (day) is shown with white and black represents lights off (night) for each day.

For the three parasitoid species tested, marking did not affect locomotor activity. Based on the visual plots, marked *G. brasiliensis* appeared to have lower or more erratic activity than unmarked individuals (Figure 4). Though there were some differences in intensity of activity on specific days, these were not statistically significant after adjusting for false discovery rates (Table S1).

### 3.6. Field Mark–Release–Recapture of T. japonicus

Out of the ~9150 wasps released at the hazelnut farm in 2022, only 14 marked individuals were recaptured. However, we did not capture any unmarked individuals identified as *T. japonicus*, indicating that poor mark retention did not lead to reduced counts. Wasps were found at traps at a maximum distance of 100 m from the release point with most within 50 m of the release site. The last captures of marked specimens were three weeks post-release, verifying the long-term viability of the marker under field conditions.

Although many more marked wasps (~19,000) were released in 2023, the recaptures were even lower, with only six marked individuals recovered on sticky traps. Again, we did not capture any unmarked *T. japonicus* on traps. The furthest captures were 100 m from the crop release point, one capture 50 m from the natural area release, and the remaining captures were within 25 m of their respective releases (two near each release point). Analysis of the wasps for the presence of the two protein markers revealed that only four out of the six individuals were positive for either protein mark. All four of these specimens had protein marks matching the release treatment they were recaptured closest to (e.g., rabbit IgG-marked specimens were recaptured closer to the field center).

## 4. Discussion

We show that fluorophore taggants can be used as an effective marker for small parasitoids, which is in agreement with their demonstrated efficacy on other arthropods [19,21,22,31]. Our study demonstrates a minimal impact on parasitoid fitness, which is an advantage over other visual markers that may inhibit the movement of these small insects [13]. Throughout fitness assessments (survivorship, parasitism, take-off, activity), the fluorophore-marked parasitoids performed equally as well as unmarked individuals. Overall, we found this marker easy to apply and detect without significantly impacting the natural behaviors observed in our laboratory studies.

Although several studies have shown the potential efficacy of this fluorophore taggant on different arthropods, many of these have not examined the marker retention past the first few days. We found that cartax green is long-lasting for all species tested, being detectable on nearly 100% of individuals up to three weeks after marking, a time frame also demonstrated for mosquitos by Faiman et al. [22]. However, we found that the magenta marker was an ineffective long-term marker for the parasitoids. Specifically, most individuals did not have visible magenta markers after only eight days, compared to the nearly three times longer retention of cartax green. Although several colors remained visible long-term for mosquitos [22], Hagler et al. [21] also found the non-green colored fluorophores to be less effective on some arthropods.

Marked individuals were easy to spot on yellow sticky cards without the need to remove any specimens even weeks after marking, which saved labor compared to many other marking techniques that require individual specimen analysis [19]. A bonus for MRR studies is avoiding the need for specialized taxonomic identification. Training is often required to distinguish parasitoid species of interest from other members of the same family or genus. By inspecting marked individuals on the trap, there is no need to confirm species identification of any suspected recaptures as those from the experiment can be quickly assessed directly on the card. Even in the case of using multiple treatments through secondary marking (e.g., protein marking), specimens of interest can be quickly identified with a visual assessment of fluorescent markers, removing the need to identify specimens prior to ELISA analysis to avoid analyzing false recaptures.

We did find some nuances in our survival data. Interestingly, the effects of marking on longevity were not consistent across species, with *T. japonicus* appearing to live longer when marked, while *P. vindemiae* had a shorter lifespan. Both species had several individuals of both treatments that lived over 20 days, which may be longer than many field populations of such small parasitoids would live [32]. This indicates that marked individuals should still live relatively long lives sufficient for MRR experiments. Additionally, the initial die-off of individuals in the experiment was similar for marked and unmarked individuals of both species. Thus, we suspect that marking was not a primary cause of mortality, even for *P. vindemiae*, which showed a significant difference in survival. We take this to be some level of stochasticity and not necessarily a direct negative effect of marking on the longevity of parasitoids, especially given the discrepancy in results between parasitoid species.

Our study also provides the first field evidence of cartax green fluorophore viability for MRR research. We successfully released and recaptured marked *T. japonicus* at two field sites over two summers. Unfortunately, our capture rates were very low, which is typically a limiting factor for MRR experiments. Given that we did not capture any unmarked *T. japonicus*, poorly marked recaptures were likely the result of trapping effectiveness rather than individuals failing to retain the mark. In fact, we captured two marked *T. japonicus* in 2022 more than three weeks after the final release. With traps having been collected weekly, these individuals were foraging for a minimum of two weeks in the field before capture and were still detected visually on the sticky card.

In 2023, the wasps were double-marked with proteins and fluorophore. Only four out of the six recaptured wasps had detectable levels of protein, whereas all six contained the fluorophore. This, in combination with the lack of unmarked recaptures, suggests that cartax green is better suited for the MRR of parasitoids given the high retention and ease of detection compared to the established protein marking technique. However, it is possible that the application of cartax green inhibited the detection of the proteins, but preliminary experiments suggested this was not the case.

This is the first study to date that provides evidence on the longevity or short-term dispersal of *T. japonicus* in the field. Our last recaptures in 2022 show that *T. japonicus* can survive at least two weeks in the field, and these individuals were already several days old at the time of release. Females of *T. japonicus* are known to live for several months or more under laboratory conditions [33,34] but its lifespan would expectedly be shorter under field conditions, as demonstrated with another small parasitoid [32]. Like many parasitoid species, *T. japonicus* survives longer when provided carbohydrate sources in the laboratory (e.g., floral nectar) and perishes quickly without them [33]. Our finding of *T. japonicus* two weeks post-release suggests that they fed on carbohydrates in the field. Since our study site was a monoculture hazelnut field with no observed flower sources, we suspect that *T. japonicus* fed on other resources provided by hazelnut trees or their associated insect community (e.g., filbert aphid honeydew).

Of interest were the two females captured more than two weeks post-release, both captured within 50 m of the release point, and the maximum distance of any recaptured *T. japonicus* was only 100 m from the release site. This suggests that the immediate dispersal ability of *T. japonicus* is rather low, and a large number of release points would be needed to expect significant augmentative biological control effects on populations of its target host, *H. halys* (brown marmorated stink bug; BMSB). We cannot rule out the possibility of some individuals traveling farther that were simply not captured, given that our recapture rates were exceptionally low (~0.2% in 2022 and ~0.04% in 2023). Our current data reveal the minimum ability of *T. japonicus* in that they can disperse at least 100 m in a two-week period.

The reason for the large difference in the recapture rate of *T. japonicus* between 2022 and 2023 is unknown. We present a few suggestions that could explain this difference. First, there was a notable difference in the populations of BMSBs between the two sites. While both sites had at least some portions dedicated to hazelnuts, the site in 2022 had easily observable populations of BMSBs throughout the site. Though we did not sample for BMSBs, egg masses, nymphs, and adults were observed on most trees at the 2022 site. In contrast, no BMSBs were observed at the 2023 site. Ecological theories on parasitoid patch foraging suggest that wasps experiencing frequent host encounters are more likely to remain in the patch than those experiencing few encounters [35]. As such, wasps at the 2022 site may have remained in the field longer and foraged more actively than at the 2023 field site. Therefore, it is likely that the parasitoids released in 2023 left the immediate foraging areas in search of hosts. Second, climatic differences could influence the dispersal ability or survival of *T. japonicus*. Furthermore, our study speaks to the overall ineffectiveness of yellow sticky cards for capturing *T. japonicus*. Despite the high number of released individuals (outweighing most other releases), we captured exceptionally few individuals, including no naturally occurring populations. Sticky cards in previous studies have demonstrated effective capture of *T. japonicus* [36,37], but these studies often have traps placed in heavily infested BMSB hosts such as tree of heaven, *Ailanthus altissima* (Simaroubaceae). It is likely that yellow sticky cards are not particularly attractive to *T. japonicus* and instead are capable of capturing individuals that happen to be nearby when placed in prime host-aggregated areas. This idea is also supported by the higher detection rates of sentinel egg masses (an attractive source) compared to yellow sticky cards (likely passive capture) in other studies [38,39].

## 5. Conclusions

We conclude that cartax green fluorophore is an effective marker for small parasitoids. This marker takes the positive aspects of protein marking (e.g., ease of application, minimal fitness effect) and improves the ease of detection by avoiding complex and time-consuming ELISA tests, allowing minimal training to detect marked specimens, and eliminating the need to remove individuals from sticky traps. As has been demonstrated in many previous studies, successful recapture rates remain the limiting factor in MRR research with these small insects. Improvements in trap designs that are more attractive to parasitoids (in this case *T. japonicus*) would greatly benefit MRR research on small parasitoids. Further field studies are needed to assess the effectiveness of the marker with other insects, but we have joined the increasing number of publications supporting the use of this marker across a wide range of taxa.

## Data Availability

The original data presented in the study are included in the article as Supplementary Materials. Further inquiries can be directed to the corresponding author.

## Supplementary Materials

The following supporting information can be downloaded at https://www.mdpi.com/article/10.3390/insects15070487/s1, Figure S1: Map of 2022 release site; Figure S2: Map of 2023 crop release site; Figure S3: Map of 2023 natural area release site; Figure S4: Persistence of fluorophore marker; Figure S5: Wing marking visibility; Figure S6: Abdomen marking visibility; Figure S7: Head marking visibility; Figure S8: Thorax marking visibility; Table S1: Activity pattern pairwise comparisons; Data file S1.
