# Supplementary material for: An Effective Fluorescent Marker for Tracking the Dispersal of Small Insects with Field Evidence of Mark–Release–Recapture of Trissolcus japonicus
Source: Insects. 2024 Jun 29;15(7):487. doi: 10.3390/insects15070487 (PMC11276981; doi:10.3390/insects15070487)
Supplement: Supplementary file 1 [file insects-15-00487-s001.zip › insects-3068042-supplementary materials/insects-3068042-supplementary.pdf]

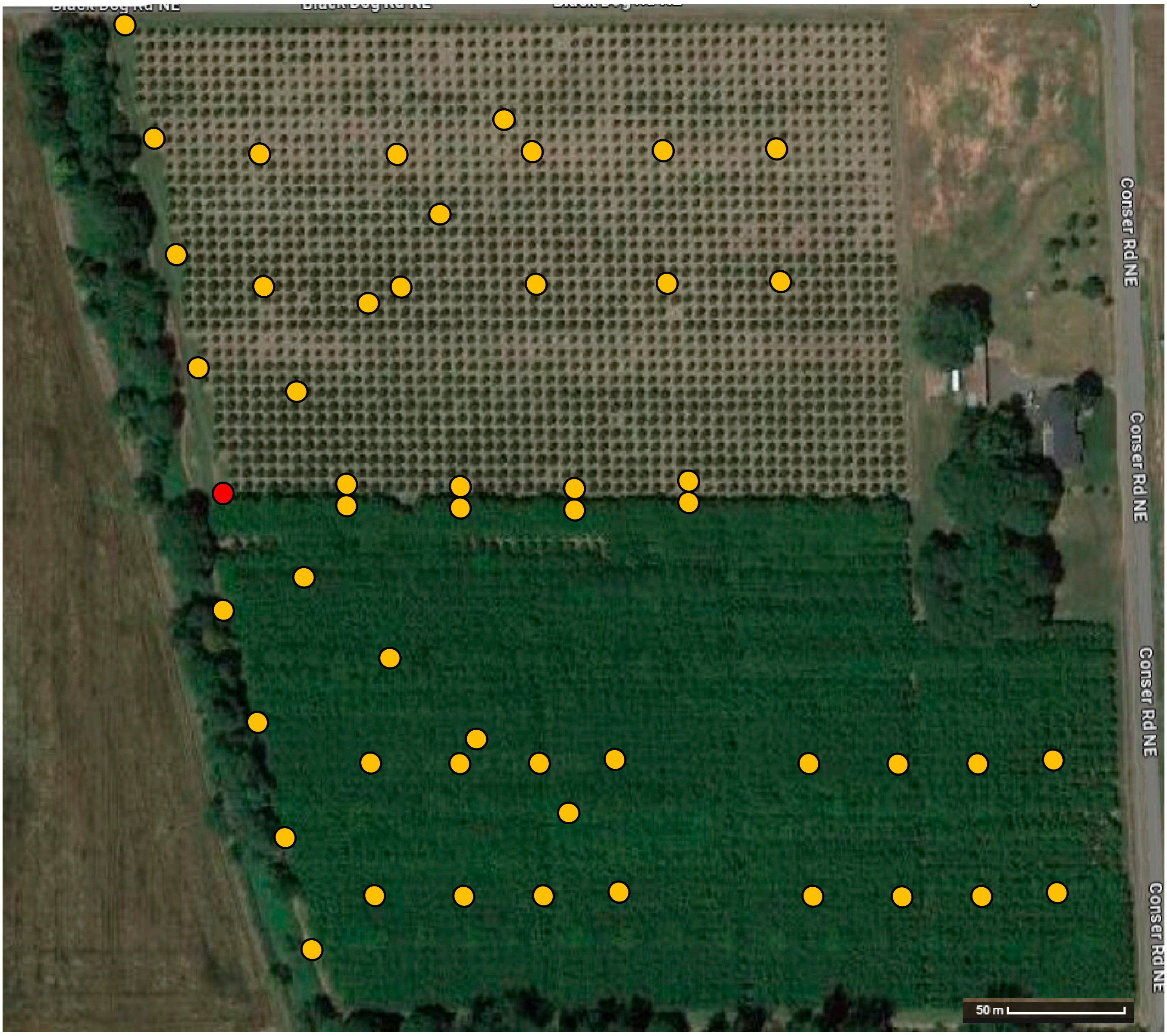

Figure S1. Map of 2022 hazelnut farm release site. Release site is shown in red and individual sticky trap locations are shown in yellow.

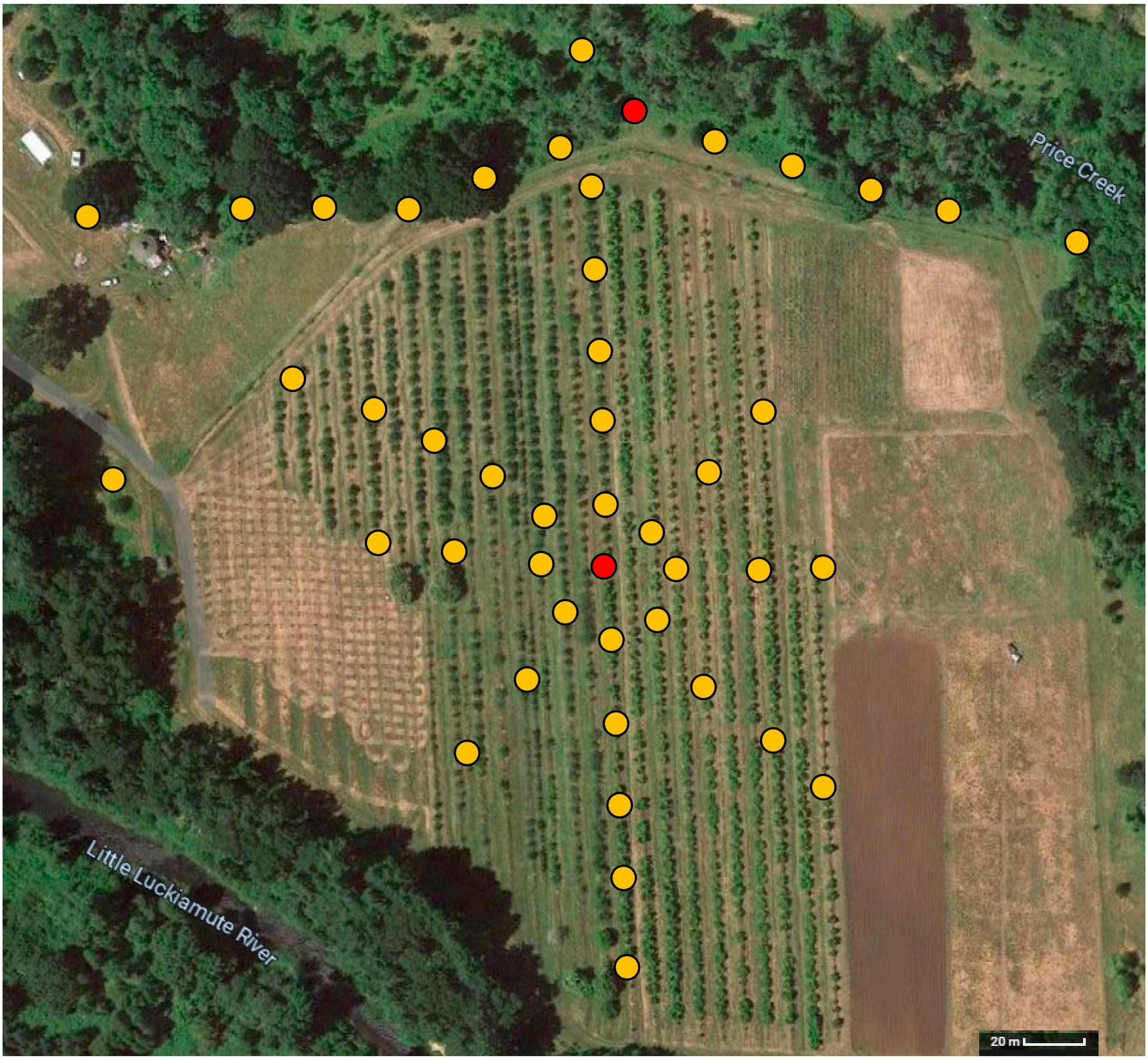

Figure S2. Map of 2023 release field site showing crop release and traps. Red circles represent locations of release sites and yellow circles depict location of individual sticky traps.

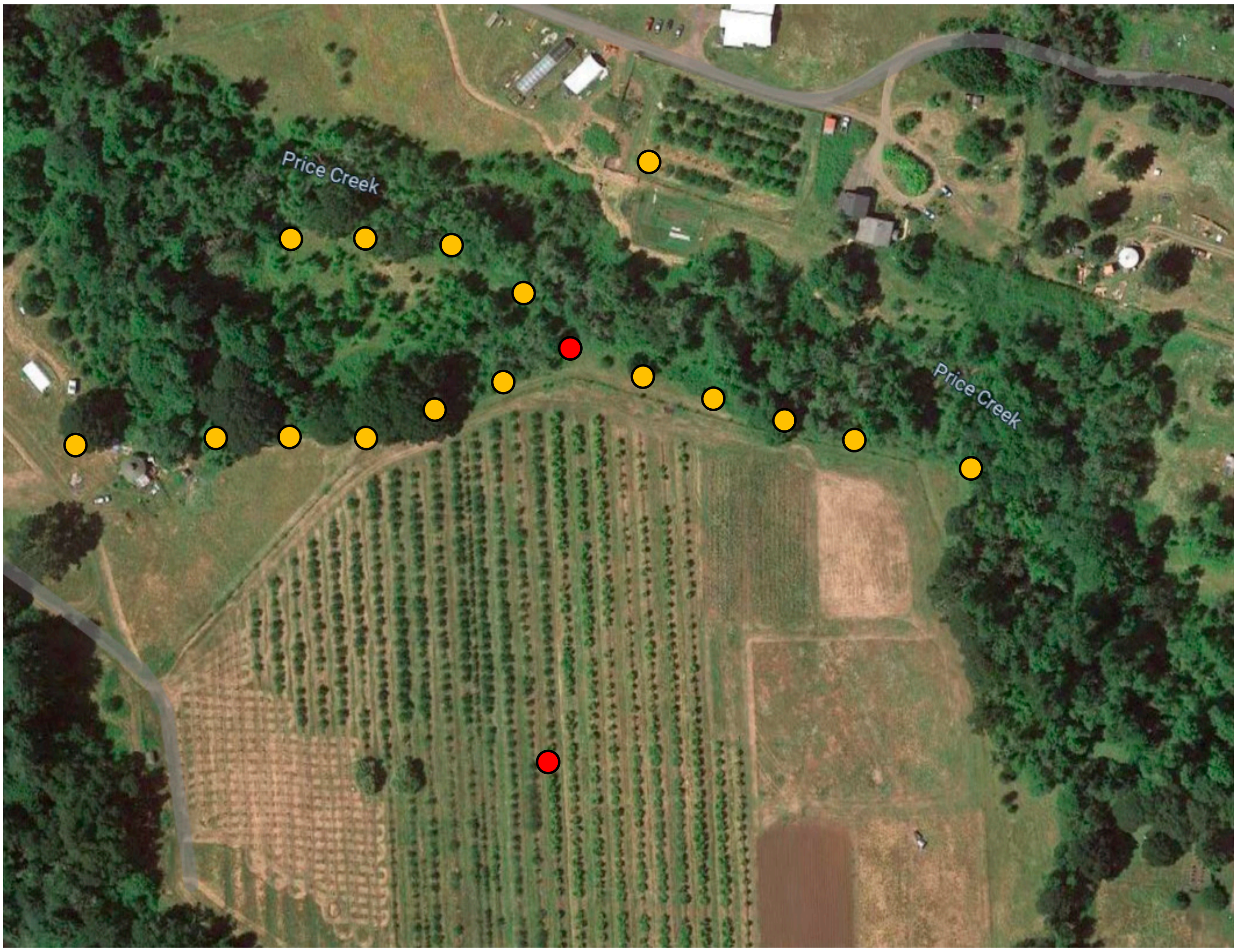

Figure S3. Natural area release site and traps from 2023 field site. Red dots represent release sites and yellow dots represent sticky traps

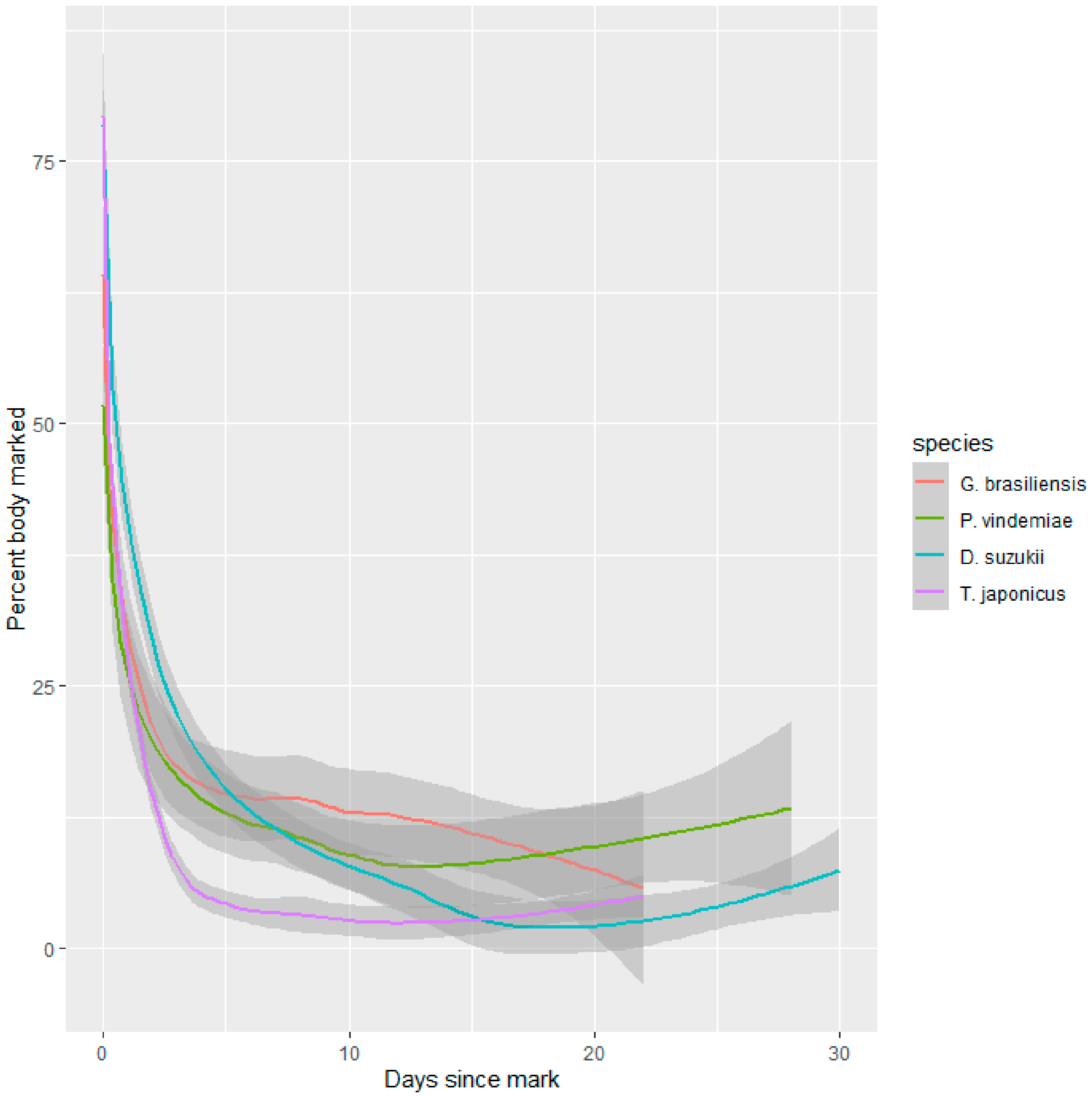

Figure S4: Persistence of fluorophore marker. Line colors represent each of the four different species testing with the marker. Percentage of body covered was converted from the averages of the visual scoring system used to assess marked individuals.

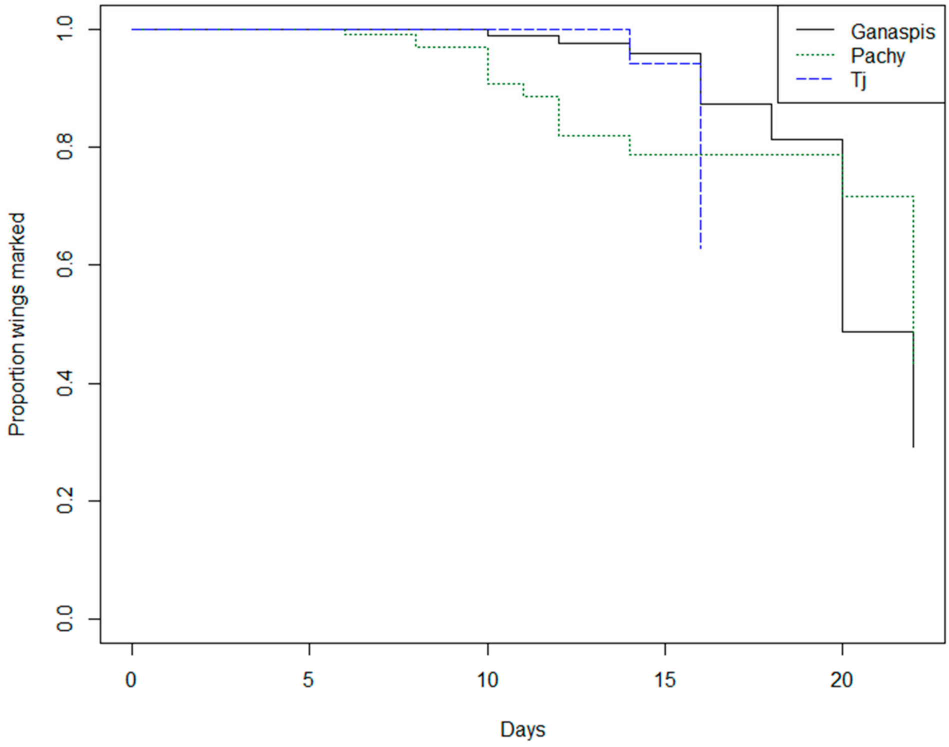

Figure S5: Wing marking visibility for the three parasitoid species based on days since marking

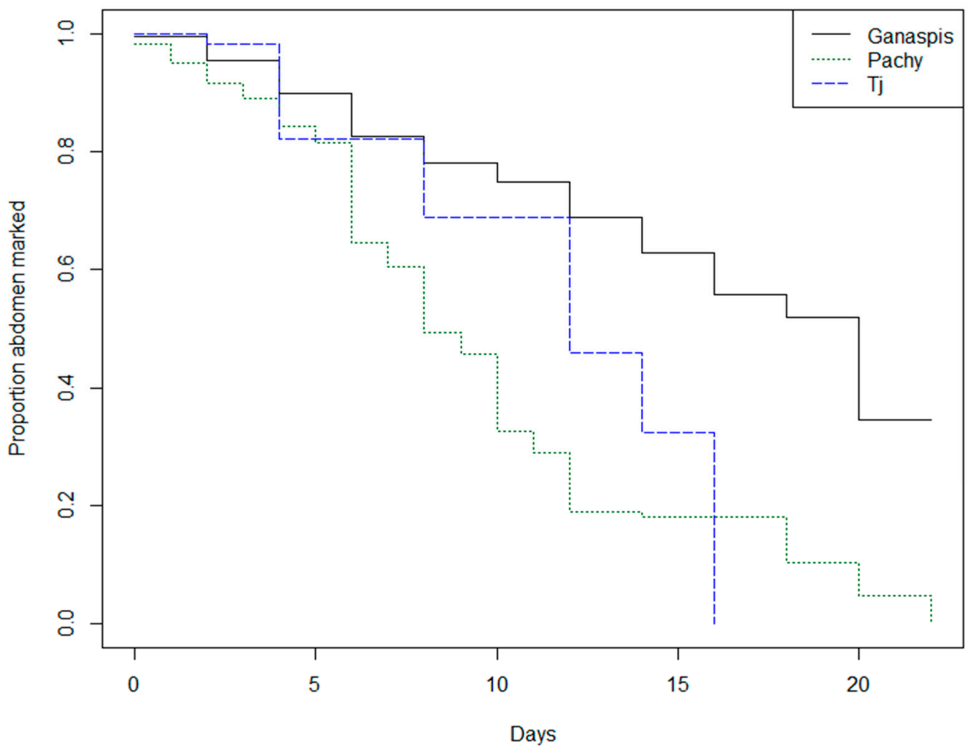

Figure S6: Abdomen marking visibility for the three parasitoid species based on days since marking

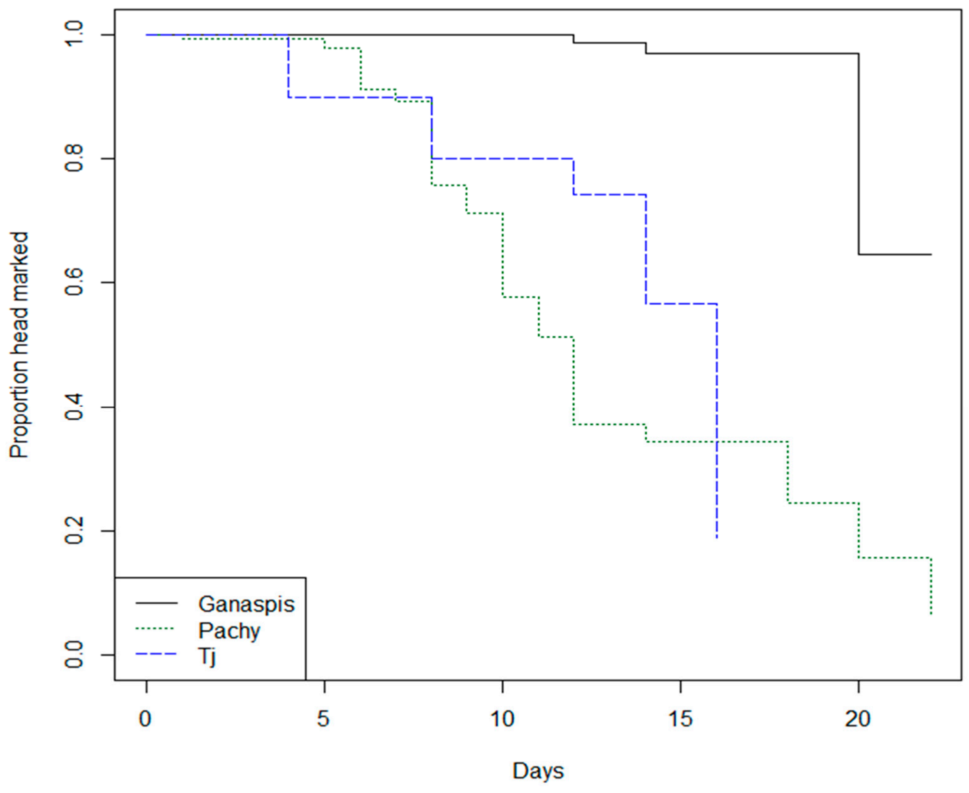

Figure S7: Head marking visibility for the three parasitoid species based on days since marking

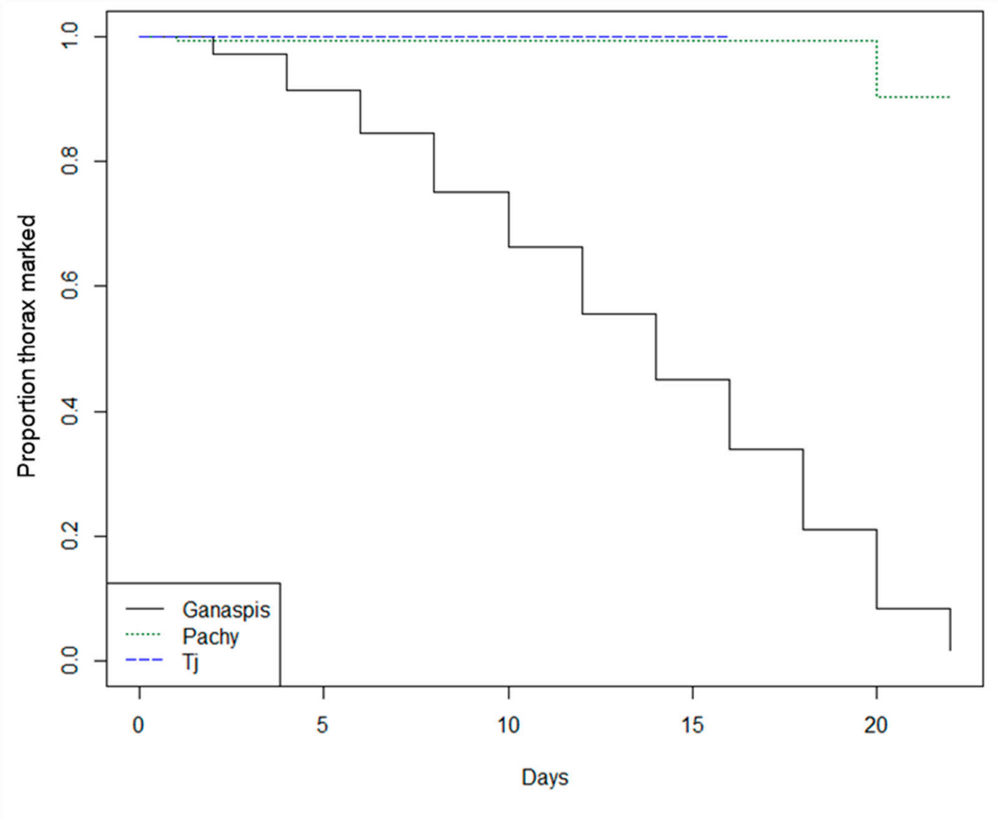

Figure S8: Thorax marking visibility for the three parasitoid species based on days since marking

Table S1: Activity pattern pairwise comparisons. Adjusted p-values for pairwise comparison of marked and unmarked parasitoid activity patterns for each day of the experiment. Day 0 is the first full day of activity measurement. Columns show the different activity responses (Daily mean activity, intensity, total activity) for each of the parasitoid species tested (Tj = T. japonicus, Pv = P. vindemiae, Gb = G. brasiliensis).

	Day	Daily.mean.Tj	Intensity.Tj	Total.Tj	Daily.mean.Pv	Intensity.Pv	Total.Pv	Daily.mean.Gb	Intensity.Gb	Total.Gb
1	0	0.8863	0.8328000	0.8863	0.5909167	0.7364000	0.5909167	0.3588	0.1934333	0.3588
2	1	0.8863	0.6598667	0.8863	0.6057000	0.6319833	0.6057000	0.3588	0.1934333	0.3588
3	2	0.8863	0.6598667	0.8863	0.5909167	0.6319833	0.5909167	0.3588	0.1934333	0.3588
4	3	0.8863	0.8328000	0.8863	0.5909167	0.6319833	0.5909167	0.3588	0.4855667	0.3588
5	4	0.8863	0.8328000	0.8863	0.5909167	0.1038800	0.5909167	0.3588	0.4855667	0.3588
6	5	0.8863	0.6598667	0.8863	0.5909167	0.3446333	0.5909167	0.3588	0.5026000	0.3588
7	6	0.8863	0.8328000	0.8863	0.5909167	0.3446333	0.5909167	0.3588	0.4855667	0.3588
